# Protective CD8+ T-cell responses to cytomegalovirus driven by rAAV/GFP/IE1 loading of dendritic cells

**DOI:** 10.1186/1479-5876-6-56

**Published:** 2008-10-05

**Authors:** Yuefei Yu, Petra Pilgrim, Juqiang Yan, Wei Zhou, Marjorie Jenkins, Nicoletta Gagliano, Klaus Bumm, Martin Cannon, Aldo Milzani, Isabella Dalle-Donne, W Martin Kast, Everardo Cobos, Maurizio Chiriva-Internati

**Affiliations:** 1 Division of Hematology & Oncology, Texas Tech University Health Sciences Center and Southwest Cancer Treatment and Research Center, Lubbock, TX, USA; 2 Departments of Internal Medicine and Obstetrics & Gynecology, and the Laura W. Bush Institute for Women's Health and Center for Women's Health and Gender-Based Medicine, Texas Tech University Health Sciences Center, Amarillo, TX, USA; 3 Department of Human Morphology, University of Milan, Italy; 4 Department of Otorhinolaryngology, Head & Neck Surgery, University of Erlangen-Nuremberg, FAU Medical School, Erlangen, Germany; 5 Department of Microbiology and Immunology, University of Arkansas for Medical Sciences, AR, USA; 6 Department of Biology, University of Milan, Milan, Italy; 7 Departments of Molecular Microbiology & Immunology and Obstetrics & Gynecology, Norris Comprehensive Cancer Center, University of Southern California, Los Angeles, CA, USA; 8 Kiromic, Inc., Lubbock, TX, USA

## Abstract

**Background:**

Recent studies demonstrate that recombinant adeno-associated virus (rAAV)-based antigen loading of dendritic cells (DCs) generates *in vitro*, significant and rapid cytotoxic T-lymphocyte (CTL) responses against viral antigens.

**Methods:**

We used the rAAV system to induce specific CTLs against CVM antigens for the development of cytomegalovirus HCMV) gene therapy. As an extension of the versatility of the rAAV system, we incorporated immediate-early 1 (IE1), expressed in HCMV. Our rAAV vector induced a strong stimulation of CTLs directed against the HCMV antigen IE1. We then investigated the efficiency of the CTLs in killing IE1 targeted cells.

**Results:**

A significant MHC Class I-restricted, anti-IE1-specific CTL killing was demonstrated against IE1 positive peripheral blood mononuclear cells (PBMC) after one, *in vitro*, stimulation.

**Conclusion:**

In summary, single PBMC stimulation with rAAV/IE1 pulsed DCs induces strong antigen specific-CTL generation. CTLs were capable to lyse low doses of peptides pulsed into target cells. These data suggest that AAV-based antigen loading of DCs is highly effective for generating human CTL responses against HCMV antigens.

## Background

Over the past few years, overwhelming evidence has come to light that inflammation hidden deep in the body is a common source of heart attacks, even when clogging of the arteries by plaque is minimal [1]. A leading cause is infection by various microbes, in particular, the human cytomegalovirus (HCMV), which historically has been linked to heart/arterial disease [2-15]. Existing drugs for the treatment or prevention of HCMV disease are only partially effective, have a variety of side effects, and may fail because of drug resistant mutations [12,16,17]. An effective HCMV gene therapy would provide a great medical benefit and would also result in annual savings in the cost of caring for persons with HCMV disease. Although immunotherapeutic interventions are promising, current treatments to induce strong immune responses against HCMV are still inadequate. In order to develop a new method to induce strong immune responses against HCMV, we used the rAAV-based antigen loading of DCs to generate significant and specific CTL responses against HCMV antigens. Several HCMV proteins have been shown to serve as target antigens for the class I-restricted CD8+ T cell responses against HCMV, including the major immediate-early protein (IE) [18], glycoprotein B (gB) [18], and non-envelope structural virion proteins, such as the matrix protein pp65 [19]. Among the IE proteins, two nuclear regulatory phosphoproteins, IE1 and IE2, are the first and most abundantly expressed proteins and are synthesized by differential splicing from the same complex overlapping transcription unit within the major IE (MIE) locus [20]. Early analyses of the CTL response in seropositive individuals have suggested that the 72-kDa immediate-early protein IE1 was a dominant target for CD8^+ ^CTLs [18]. IE1 is the major protein produced in the immediate-early phase of the human HCMV replication cycle and has been shown to be target for CD4^+ ^and CD8^+ ^T cells [21]. IE1 was the first gene product identified to elicit CTL responses in mice [22]. The role of IE1-recognizing CD8+ T cells will be an interesting subject to study. DCs are professional antigen presenting cells that are critical to prime a cellular immune response [12,23-25]. There is evidence of several protocols for loading DCs, based on the use of tumor antigens such as peptides, lysed tumors, whole proteins, and genes expressed on plasmids or viral vectors [26,27]. These new technologies permit *in vitro *manipulation of DCs for clinical studies [12,28,29].

Recent studies demonstrate that recombinant rAAV-based antigen loading of DCs generates significant and rapid CTL responses *in vitro *[12,19,30]. rAAV has been widely studied in applications to transduce DCs. rAAV lacks viral coding sequences, therefore the transduced DCs only express antigen proteins and not viral proteins [31]. Further, rAAV does not elicit an immune response in its host, therefore there is no secondary inflammation in the host due to rAAV [31].

In the present study, IE1 genes were cloned into AAV to test the ability of r-AAV loading of DCs to generate specific CTL responses against IE1 positive cells.

## Methods

### Cell culture and patients material

The HEK293 cells were maintained and propagated in complete DMEM supplemented with penicillin and streptomycin (Mediatech Inc., Herndon, VA) and 10% FBS (Gemini Bio-Products, West Sacramento, CA). Autologous peripheral blood mononuclear cells (PBMCs) and were obtained from 3 female HLA-A2 restricted healthy donors. All of the clinical materials were obtained with the patient's consent and approval by the local ethics committee.

### Constructing the AAV/IE1 genome and generation of virus stocks

The AAV/IE1 genome was constructed as a plasmid as previously described [28,30]. Briefly, the IE1 gene was amplified by PCR from plasmid pCGN-IE1, which was kindly provided by Dr. Thomas Shenk at the Department of Molecular Biology, Princeton University. PCR amplification for IE1 was carried out using the following primer pair: upstream, 5'-GGTACCATGGAGTCCTCTGCCAAGA-3'; downstream, 5'-CTCGAGGACCTTGTACTCATTACACATTG-3'. AAV/IE1 virus stocks were generated using complementary plasmids ins96-0.8 or pSH3, using HEK293 cells as described previously [28,30,32]. Lysates of HEK293 cells were used as virus-negative controls for mock infections.

### Immunofluorescence

HEK293 cells were spun in a cytospin column (5 × 10^4 ^cells/slide), fixed with SlideRite (Fisher, USA), and air dried overnight. Each sample was permeabilized (P) in PBS 1×/0.1% Triton X-100 for 15 minutes at 4°C not permeabilized (NP). Results were analyzed using an Olympus IX71 inverted microscope equipped with a Fluoview 300 confocal laser system.

### Real-time PCR for virus stock titration

The titer of virus stocks was determined by real-time PCR as previously described [32]. Briefly, we used the plasmid AAV/IE1 for the real-time PCR standards, respectively. Concentration was measured by absorbance at 260 nm.

### Generation and infection of monocyte-derived DCs

Autologous DCs (2 × 10^5 ^adherent monocytes) were generated and infected (0.5 mL virus [10^9 ^eg/mL]) as previously described [28,30]. Recombinant granulocyte macrophage-colony-stimulating factor (GM-CSF) (R&D Systems, Minneapolis, MN, USA), at a final concentration of 800 IU/mL, was included in the medium throughout the culture. To induce monocytes into DCs, human interleukin-4 (IL-4) (R&D Systems, Minneapolis, MN, USA) at 1000 IU/mL was added on day 3, after infection.

### Generation of autologous 1E1-positive target cells

Non-adherent PBMCs, isolated from healthy donors, were infected with AAV/IE1 virus at a multiplicity of infection of 100, 4 days before the ^51^Cr release assay.

### Lipofection using DOTAP

The recombinant IE1 protein was made as previous described [33]. Lipofection was performed using the cationic liposome-mediated transfection reagent, DOTAP (Roche Diagnostics, Indianapolis, IN). IE1 protein was mixed with the DOTAP reagent and serum-free media at ratios following the manufacturer's recommendations. The cells were then incubated in serum-free media containing the lipofection mix for 4–6 hours. Final IE1 concentration was 100 nM for the DCs and PBMCs. After 4–6 hours of incubation, serum-supplemented DMEM was added to cells. After 24 hours, all of the lipofection media was replaced with fresh growth media for cells.

### Generation and testing of 1E1-specific CTLs

CTL were generated from 3 normal donors (HLA matched). Experiments were performed in quadruplicate (experiments were preformed 4 times independently with different ratios of responders to DCs from 5:1; 10:1; 20:1; 40:1 data not provided) [23,24]. For each experiment, the non-adherent PBMCs were washed and re-suspended in AIM-V at 10 to 20 × 10^6 ^cells per well in 6-well culture plates with AAV/IE1-loaded autologous DCs (optimal ratios of responders to DCs from 20:1). The cultures were supplemented with GM-CSF (800 U/mL) and recombinant human IL-2 (10 U/mL). After 7 days of co-culture, the cells were used for cytotoxicity assays in a 6-hour ^51^Cr assay, as previously described [16,23,24]. To determine the CTLs' HLA restriction, HLA-class I (W6/32) of antibodies, at a concentration of 25 μg/mL, were pre-incubated with the target cells for 30 minutes before addition of the stimulated T-cells. K562 cells were used as targets to observe natural killer (NK) cell activity. In all of these CTL killing assays, spontaneous release of chromium never exceeded 25% of the maximum release [23,24].

### Flow cytometry analysis

This protocol was adapted from that described by Pala et al. and modified [24,28]. Cell surface marker analysis of T cells and DCs was conducted using fluorescence-activated cell scanning (FACS) (FACScan; BD Biosciences-PharMingen, Franklin Lakes, NJ), as described previously [24,28].

### Statistical analysis

All results are expressed as mean ± SD. Data were analyzed using nonparametric analysis of variance (ANOVA). Differences were considered significant if *P *< 0.05.

## Results

### Construction of AAV/IE1 Recombinant Viruses

**Figure 1:**
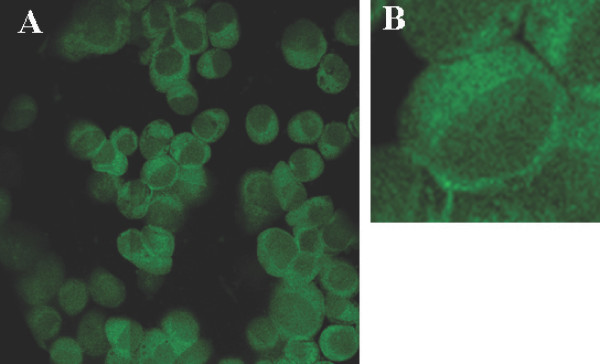
**Immunofluorescence on HEK293 cells. **Microphotographs show fluorescent labeling for AAV/IE1 (A, B) in HEK293 cells. A: original magnification: 20×; B: original magnification: 63×.

The goal of this study was to determine whether rAAV-based gene loading of IE1 genes into DCs could elicit a significant CTL response against IE1-positive target cell lines. This was the first time that the gene encoding IE1 was inserted into the AAV vector. First, the IE1 gene was amplified by PCR from plasmid pCGN-IE1. The IE1 cDNA obtained from pCGN-IE1 was inserted into the gutted AAV vector to generate AAV/IE1 as described in the materials and methods section. Figure 1A shows a structural map of the AAV/IE1 vector. In this vector, the IE1 gene was expressed from the AAV p5 promoter, which is known to be active in DCs [31]. After rAAV vector generation, we evaluated their ability to infect HEK293 cells. The rAAV-vector infected cells expressed the target antigens, as confirmed by immunofluorecence labeling, which showed the expression of IE1 transduced HEK293 cells. (Figure 1)

### Titration of AAV/IE1 virus stocks using real-time PCR assays

**Figure 2:**
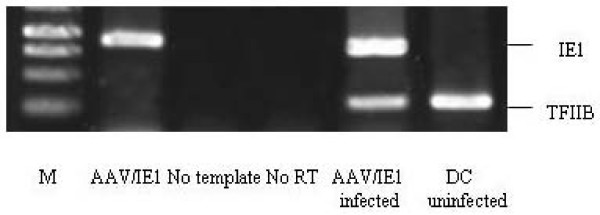
**Virus stock titers.** DNA extracted from the purified virus of AAV/IE1 was used as the template of PCR. The DNA from 1000 μl, 500 μl and 250 μl purified virus was tested, respectively. We used three blank wells, with water, as negative controls. EG = encapsulated genomes.

Virus stock titers were determined by real-time PCR (Figure 2). We assessed the linearity of the real-time PCR by using a dilution row of the AAV/IE1 plasmid that would serve as standard curve in all further experiments. The obtained fragments corresponded to the expected size and no additional bands could be detected by gel electrophoresis, showing the specificity and selectivity of the PCR. We did not observe signals from the template sample in either the amplification plot or the agarose gel photograph (data not shown).

### AAV/IE1-transduced DCs express 1E1

**Figure 3:**
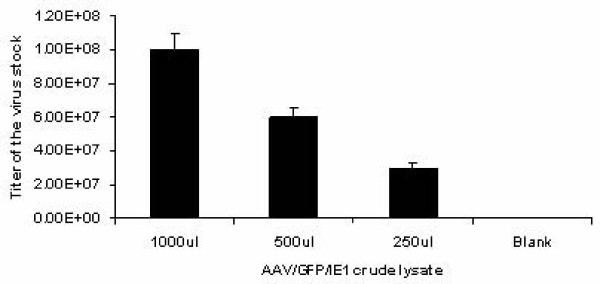
**IE1 expression in infected DCs.** Total RNA was isolated from mock-infected and AAV/IE1-infected adherent monocytes at 72 hours after infection. These samples were analyzed by RT-PCR and PCR, as indicated, for the presence of IE1 RNA. PCR product resulting from using the AAV/IE1 vector plasmids as templates was the positive controls. RT-PCR analysis for the cellular *TFIIB *mRNA was considered as further control. Note that only cDNA from cells infected with AAV/IE1 virus resulted in an appropriate RT-PCR sized product, whereas mock-infected cells did not.

Protocols for generating DCs by differentiating PBMCs usually involve the use of GM-CSF and IL-4 during adherent monocyte culturing. We modified this protocol to promote AAV vector transduction in DC precursor monocytes by treating adherent monocytes just after AAV infection with GM-CSF alone, adding IL-4 on day 3. This method allowed higher levels of AAV transduction [34]. Figure 1B shows a schematic diagram of the experimental protocol. Monocyte/DC population transduction was confirmed by measuring polyadenylated RNA expression of the AAV/IE1 transgene. At day 10, polyadenylated RNA was isolated from AAV/IE1-infected and mock-infected DC cultures. The mRNA levels were analyzed by RT-PCR for AAV/IE1 expression. A cellular housekeeping gene, *TF*_*II*_*B*, was included as a control. IE1 mRNA expression took place only in the infected DCs (Figure 3). A PCR-only control (no RT step) failed to generate a product, indicating that there was no DNA contamination in our samples.

### AAV/IE1-transduced DCs stimulated AAV/IE1-specific CTLs

**Figure 4:**
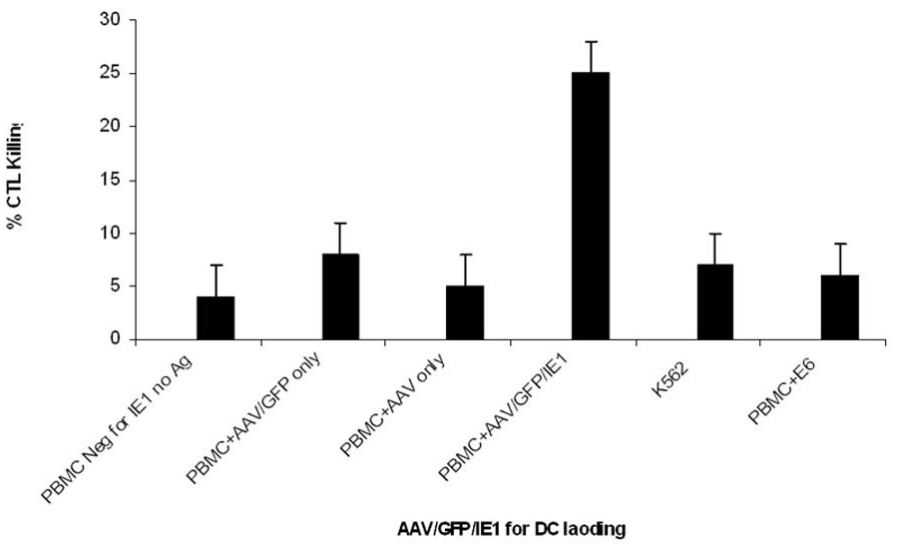
**Cytotoxicity assay. **Multiple AAV vectors for DC loading and the autologous targets generated using the IE1 sub-genes. Targets were generated by viral loading of the IE1 sub-genes into PBMC. Resulting CTL killing is shown. Note that T cells stimulated by mock-infected (no Ag) loaded DCs, AAV only-loaded DCs AAV/GFP-loaded DCs AAV/E6-loaded DCs did not kill IE1-positive targets. However, T cells stimulated by AAV/GFP/IE1-loaded DCs did kill IE1-positive target cells. These data strongly suggest high antigen-loading specificity of the CTLs generated by AAV/GFP/IE1 infection of DCs.

**Figure 5:**
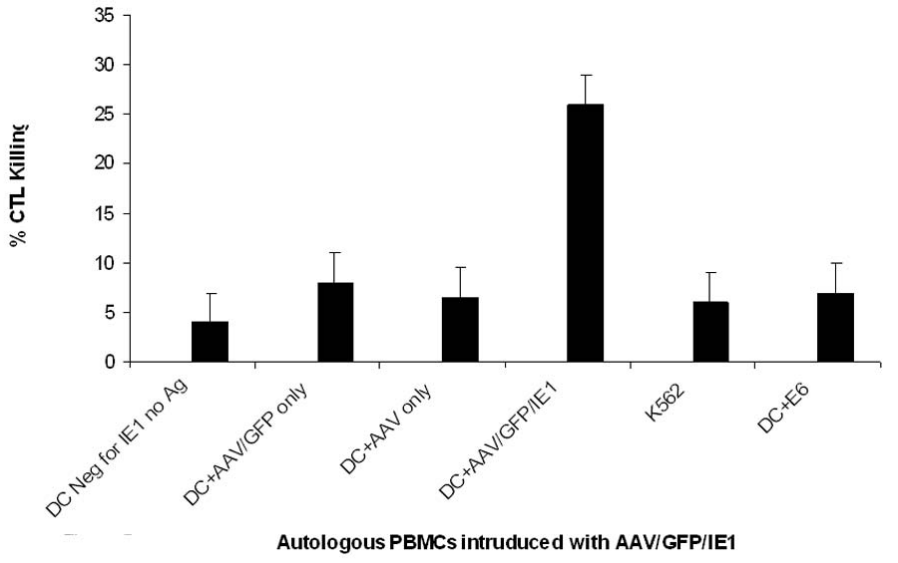
**Cytotoxicity assay.** AAV/GFP/IE1 vectors for DC loading and multiple targets generated using various vectors. Targets were generated by IE1 positive and negative vector loading into PBMC. Resulting CTL killing is shown. IE1 negative PBMCs (no Ag) and K562 cells were not killed, indicating strong antigen specificity for the CTLs generated by AAV/IE1 loading.

**Figure 6:**
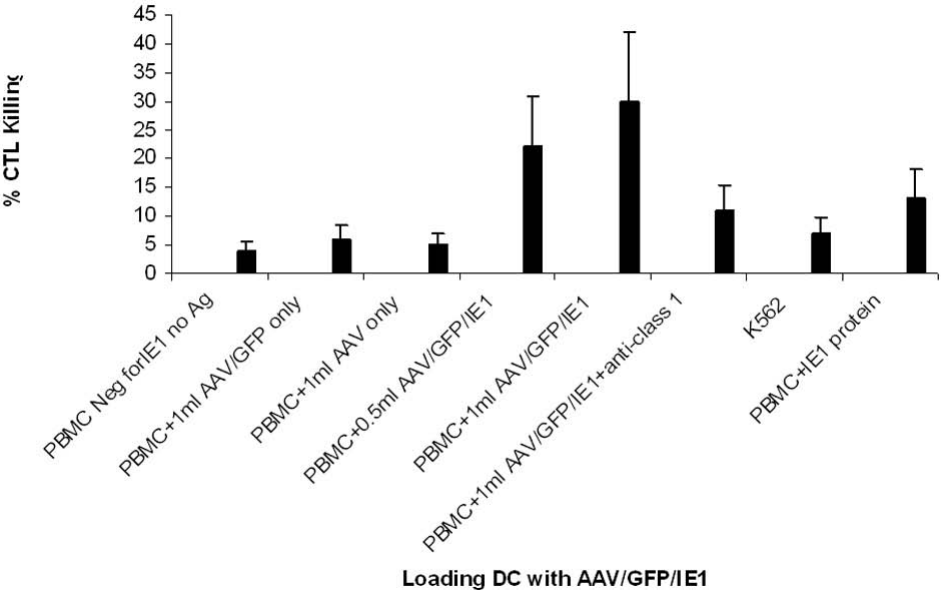
**Cytotoxicity assay.** Killing was stimulated in a dose-dependent manner. Killing activity was significantly inhibited when target cells were pre-incubated with anti-class I antibodies (P < 0.05). Similarly, the killing activity of DC transduced with AAV/GFP/IE1 showed a significant higher (P < 0.05) than IE1 protein lipofection using DOTAP did.

We analyzed the ability of the AAV/IE1 vectors to generate IE1 specific-CTLs (optimal ratio E:T; 1:20). To analyze CTL activity, we used the following 5 target cell types for the ^51^Cr release assays (Figures 4, 5, 6): 1) Autologous PBMCs. Because late B cells are only a small percentage of PBMCs, PBMCs served as an autologous, antigen-negative control; 2) PBMCs transfected with AAV/IE1 expression plasmid; 3) PBMCs transfected with AAV only and AAV/GFP, as a negative controls; 4) PBMCs transfected with E6, as a control; 5) PBMCs transfected with IE1 protein.

**Figure 7:**
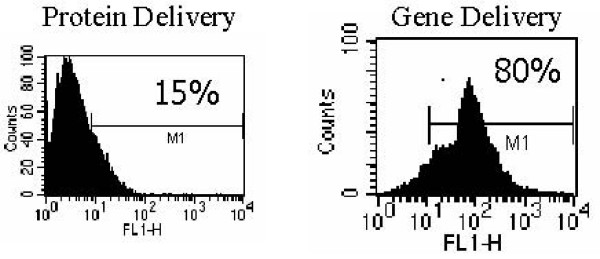
**Flow cytometric characterization.** Shown are the results of FACS analysis for the antigen delivery. Note that the use of AAV/GFP/IE1 loading DC resulted in a higher delivery effect (80%) than IE1 protein lipofected DC did (15%).

To determine the ability of AAV/IE1-transduced DCs to stimulate IE1-specific CTLs, we performed a standard 6-hour^51^Cr assay on day 7 using a 1:20 (ratio: Effector:Target) (Figure 5) using the T-cell populations primed in co-culture with the rAAV-transduced DCs [30]. We generated autologous targets by infecting donor PBMCs with AAV/IE1 virus 4 days before the CTL assay. AAV/IE1-infected PBMCs were found to express IE1 by RT-PCR analysis, whereas unaltered PBMCs and K562 cells did not express IE1 (data not shown). T-cells incubated with AAV/IE1-loaded DCs were able to kill the IE1-positive autologous target cells. These data are consistent with a strong antigen-specific CTL response. Figure 7 shows that CTL killing activity was dose-dependent and MHC class I restricted. In this experiment, 2 different doses of AAV/IE1 vector were used for DC loading and a zero virus control (PBMC only). The cytotoxicity of the stimulated T-cells directly correlated with the amount of AAV/IE1 used to load the DCs at day 0. Alternately, the addition of anti-class I antibodies significantly inhibited the killing activity (P < 0.05), suggesting that CTLs were MHC class I restricted. The CTL stimulation performed by AAV/IE1 loaded DCs was superior to the one performed by IE1 protein lipofection (P < 0.05). The negative controls (K562 and the targets pre-incubated with anti-MHC class I antibodies) did not induce significant killing activity. These data showed CTLs to be highly AAV/IE1 specific and MHC class I restricted. Figure 7 demonstrates that the use of AAV/GFP/IE1 loading DCs resulted in a higher delivery effect (80%) than IE1 protein lipofected DCs did (15%).

## Discussion

To achieve effective antivirus responses, recent emphasis has been placed on approaches that stimulate strong cellular immune responses, which are mediated by T-cells and particularly by CTLs. CTLs are believed to be the critical immune effector arm in mediating potential antivirus immunity. CD8^+ ^CTLs play a major role in protection against HCMV and in maintenance of its latency [35-38]. It has been hypothesized that antigen gene delivery into DCs [23,24] may be more efficient for generating CTLs than by antigen delivery as a lipofected, exogenous protein [23,24,28]. Although there is some controversy as to AAV effectiveness in transducing DCs and other hematopoietic cells, donor monocytes/DCs have been shown to be successfully transduced with AAV-2 [23,24,28,30]. Furthermore, in various studies, AAV has been shown to be an effective gene-delivery system for immortalized tissue-cultured cells and primary hematopoietic cells [34,39-41]. The AAV vectors were found to transduce up to 85% of DCs [12,19,23,24]. The transduced DCs displayed higher levels of CD80, CD83, CD86, and CD1a over controls. In fact, the DC-loading technique was found to be highly effective in generating significant CTLs with only one DC-T-cell co-incubation and in a time frame of only 1 week. We confirm that rAAV-infected monocytes with GM-CSF only and then adding IL-4 after 3 days induces DCs' differentiation [23,24]. Previous studies showed that rAAV-loading DCs can rapidly generate antigen-specific CTLs against viral antigens [16]. The IE1 protein has been proposed as a target for immunotherapy. The IE genes are the first ones to be expressed in the replicative cycle, and their expression does not depend on prior viral protein synthesis. Together with some virion proteins, the IE products activate viral genes and alter the infected cell to generate an appropriate milieu that favors viral replication [42]. Human cytomegalovirus (HCMV) IE1, the most abundant IE product, plays an accessory role in the IE2-mediated activation of HCMV early and late genes [43,44]. Interaction of HCMV IE1 with a number of cellular regulatory proteins has also been described previously [45]. In addition to their regulatory activities, HCMV IE1 is involved in perturbing a variety of other cellular processes, including cell cycle regulation [46,47], apoptosis [48], and cell architecture. The IE1 protein of HCMV is a major source of CD8 T-cell epitopes for HLA molecules represented in a large proportion of the human population, and plays a significant role in the control of HCMV disease [49]. The previous study led to the identification of several new classes of I MHC-restricted CTL epitopes against IE1 antigens [50]. This result was confirmed by another study in which several IE1 HLA class I epitopes were detected and no IE1 class II epitopes were identified [51].

Here we have demonstrated that rAAV-loading of DCs with IE1 can generate antigen-specific CTLs in substantial numbers, only 1 week after stimulation. Based on this and our previous studies, we hypothesize that the AAV vector causes a fundamental change in DC performance, perhaps by modifying their co-stimulatory ligand expression, resulting in more efficient generation of antigen-specific CTLs [28]. We hypothesized that the AAV/IE1 would be superior to IE1 protein in stimulating CTL killing. Our experiments show that AAV/IE1 was much more efficient in stimulating the killing of target cells than IE1 protein (P < 0.05). Our controls (Figures 5, 6, 7) show strong antigen specificity and MHC class I restriction. For example, Figure 5 shows that autologous PBMCs were not targeted for killing unless these target were preloaded with the antigen. Without loading the antigen, there is no significant killing. Furthermore, K562 cells are shown in Figures 4, 5, 6 to be insignificant targets.

This same report [51] suggested that IE1 is directly related to CTL killing and the importance of MHC class I molecules as a restriction element in HCMV. Our results prove a direct link between the IE1 protein and CTL recognition. We believe it is likely that there are multiple reasons why AAV loading of DCs is effective. One reason is the high transduction frequency we have observed. A second reason could be the increased expression of CD80, CD86, and CD40 that may also contribute to generating the robust CTL response.

## Conclusion

In summary, our results demonstrate that the delivery of IE1 antigen by an AAV vector is a good strategy for generating anti-IE1 CTLs. Our data suggest that AAV-based antigen loading of DCs is highly effective for generating a CTL response against HCMV.

## Competing interests

The authors declare that they have no competing interests.

## Authors' contributions

YY performed protein and AAV generation and all PCR experiments and drafted the manuscript. PP performed immunofluorescence experiments and drafted the manuscript. JY performed AAV generation and all PCR experiments. WZ performed AAV generation and all PCR experiments. MJ participated in study design and coordination and revised the manuscript. NG participated in the design of the study and revised and drafted the manuscript. KB participated in the design of the study and revised and drafted the manuscript. MC participated in study design and coordination and revised and drafted the manuscript. AM participated in the design of the study and revised and drafted the manuscript. IDD participated in the design of the study and revised and drafted the manuscript. WMK participated in study design and coordination and revised and drafted the manuscript. EC participated in study design and coordination and revised the manuscript. MCI carried out the study design, FACS analysis and killing assay and drafted and revised the manuscript. All authors read and approved the final manuscript.
